# Lipopolysaccharide O-Antigen Prevents Phagocytosis of *Vibrio anguillarum* by Rainbow Trout (*Oncorhynchus mykiss*) Skin Epithelial Cells

**DOI:** 10.1371/journal.pone.0037678

**Published:** 2012-05-25

**Authors:** Kristoffer Lindell, Anna Fahlgren, Erik Hjerde, Nils-Peder Willassen, Maria Fällman, Debra L. Milton

**Affiliations:** 1 Department of Molecular Biology, Umeå Centre for Microbial Research, Umeå University, Umeå, Sweden; 2 Southern Research Institute, Birmingham, Alabama, United States of America; 3 Department of Chemistry, Faculty of Science and Technology, University of Tromsø, Tromsø, Norway; University College Dublin, Ireland

## Abstract

Colonization of host tissues is a first step taken by many pathogens during the initial stages of infection. Despite the impact of bacterial disease on wild and farmed fish, only a few direct studies have characterized bacterial factors required for colonization of fish tissues. In this study, using live-cell and confocal microscopy, rainbow trout skin epithelial cells, the main structural component of the skin epidermis, were demonstrated to phagocytize bacteria. Mutant analyses showed that the fish pathogen *Vibrio anguillarum* required the lipopolysaccharide O-antigen to evade phagocytosis and that O-antigen transport required the putative *wzm-wzt-wbhA* operon, which encodes two ABC polysaccharide transporter proteins and a methyltransferase. Pretreatment of the epithelial cells with mannose prevented phagocytosis of *V. anguillarum* suggesting that a mannose receptor is involved in the uptake process. In addition, the O-antigen transport mutants could not colonize the skin but they did colonize the intestines of rainbow trout. The O-antigen polysaccharides were also shown to aid resistance to the antimicrobial factors, lysozyme and polymyxin B. In summary, rainbow trout skin epithelial cells play a role in the fish innate immunity by clearing bacteria from the skin epidermis. In defense, *V. anguillarum* utilizes O-antigen polysaccharides to evade phagocytosis by the epithelial cells allowing it to colonize rapidly fish skin tissues.

## Introduction

*Vibrio anguillarum*, part of the normal microflora on fish and in the aquatic environment, is an opportunistic pathogen that causes a fatal hemorrhagic septicemia in marine fish [1], [2]. This pathogen has had devastating impacts on aquaculture worldwide causing large economical losses. Consequently, *V. anguillarum* is one of the best studied bacterial fish pathogens. Despite the importance of this and other fish pathogens, very few direct studies have been done to characterize bacterial factors required for pathogens to colonize fish tissues [3]. Attachment to and colonization of host tissues are the first steps that many pathogens must take to cause disease.

In a study utilizing rainbow trout, *V. anguillarum* bound glycosphingolipids on the intestinal mucosa suggesting that the intestinal tract is a portal of entry into fish and a major site of colonization and proliferation [4]. Other studies suggested that the fish skin is the initial site of colonization for *V. anguillarum* and a main point of entry into the animal [5]–[7]. Recently, two studies directly investigated *V. anguillarum* infections in rainbow trout and characterized virulence factors required for colonization of the skin tissues. In the first study, a fluorescence-tagged *V. anguillarum* formed a biofilm-like structure on the fish skin and exopolysaccharide transport genes were shown to be required for formation of the biofilm [8]. A second study investigated the temporal and spatial spread of a *V. anguillarum* infection in the whole fish animal during an infection using in vivo bioluminescence imaging [9]. In this study, the skin and intestinal mucosal tissues were the first sites for bacterial colonization and proliferation; while, internal organs, such as the spleen and the kidney, were not colonized until the later stages of infection. The bacteria reached a higher cell density on the skin tissues than on the intestinal tissues and colonization of the skin tissues but not the intestinal tissues required siderophore production, the RNA chaperone Hfq, and exopolysaccharide transport genes. In addition, exopolysaccharide transport was shown to aid resistance of *V. anguillarum* to lysozyme and antimicrobial peptides, which are components of the fish skin immune defense [9].

Fish live in an aquatic environment that is rich in pathogens and the integrity of the skin epidermal mucosa is vital to form a mechanical barrier that protects fish from microorganisms in the environment [10], [11]. Fish skin consists of two basic layers, the outer epidermis and the inner dermis and the entire surface of a fish outer body is covered by the epidermal mucosa. The outer epidermis is covered by a layer of mucus, which consists of glycoproteins and is enriched with antimicrobial factors such as antibodies, complement, lysozyme, C-reactive protein, lectins, proteases, transferrin, and antimicrobial peptides [10], [11]. Hence, antimicrobial activities are vital functions associated with the fish skin tissues for disease prevention.

The epidermal outer layer of fish skin is composed predominately of epithelial cells, which may also be known by other names such as keratocytes, Malpighian cells, or filamentous cells [10]. In this study, the term epithelial cell will be used. In teleost fish, the epidermal epithelial cells are metabolically active and migrate with speeds of 5–12 µm per minute [12], [13]. Due to their fast motility, the migrating epithelial cells are thought to play a role in wound repair and protection against infectious disease [14], [15]. Immediately following skin injury, epithelial cells at the edge of the wound migrate as a network maintaining intercellular contacts and close the wound completely within a few hours to days. In addition to wound repair, Atlantic salmon (*Salmo salar*) skin epithelial cells are suggested to possess discriminatory phagocytic activities as they internalize *Carnobacterium piscicola*, *Pseudomonas fluorescens*, *Aliivibrio wodanis*, and *Aeromonas salmonicida* subsp. *salmonicida* but not *Staphylococcus intermedius* or *Moritella viscosa* [16]–[18]. Thus, skin epithelial cells are proposed to play a role in the immune defense of the skin tissues of teleost fish by removing debris and bacteria from the skin epidermis.

Nothing is known about the mechanisms used by the fish skin epithelial cells to internalize bacteria selectively or how bacteria such as *V. anguillarum* that do colonize the skin tissues may evade this host immune response. This study aimed to identify bacterial components of *V. anguillarum* that are required to evade phagocytosis by the epithelial cells. Using live-cell and confocal 3D-imaging microscopy, single, migrating rainbow trout skin epithelial cells were shown to phagocytize bacteria. Moreover, *V. anguillarum* utilized lipopolysaccharide (LPS) O-antigen to evade the phagocytosis by the epithelial cells. Using *in vivo* bioluminescence imaging, mutants defective in O-antigen polysaccharide transport failed to colonize rainbow trout skin even though they did colonize the intestinal tissues suggesting that the epithelial cells cleared the mutant strains from the skin tissues preventing colonization. The O-antigen transport mutants were also less resistant to antimicrobial components associated with the skin epidermal mucosa compared to the wild type. Thus, these newly identified roles for the O-antigen polysaccharide in *V. anguillarum* provide protection during the early stages of infection so that the bacteria may colonize and proliferate rapidly on the fish skin. In addition, rainbow trout skin epithelial cells were shown to phagocytize bacterial cells providing the epithelial cells with the capacity to play a major role in the immune defense of the fish.

## Results

### *Vibrio anguillarum* evades internalization by rainbow trout skin epithelial cells

**Figure 1:**
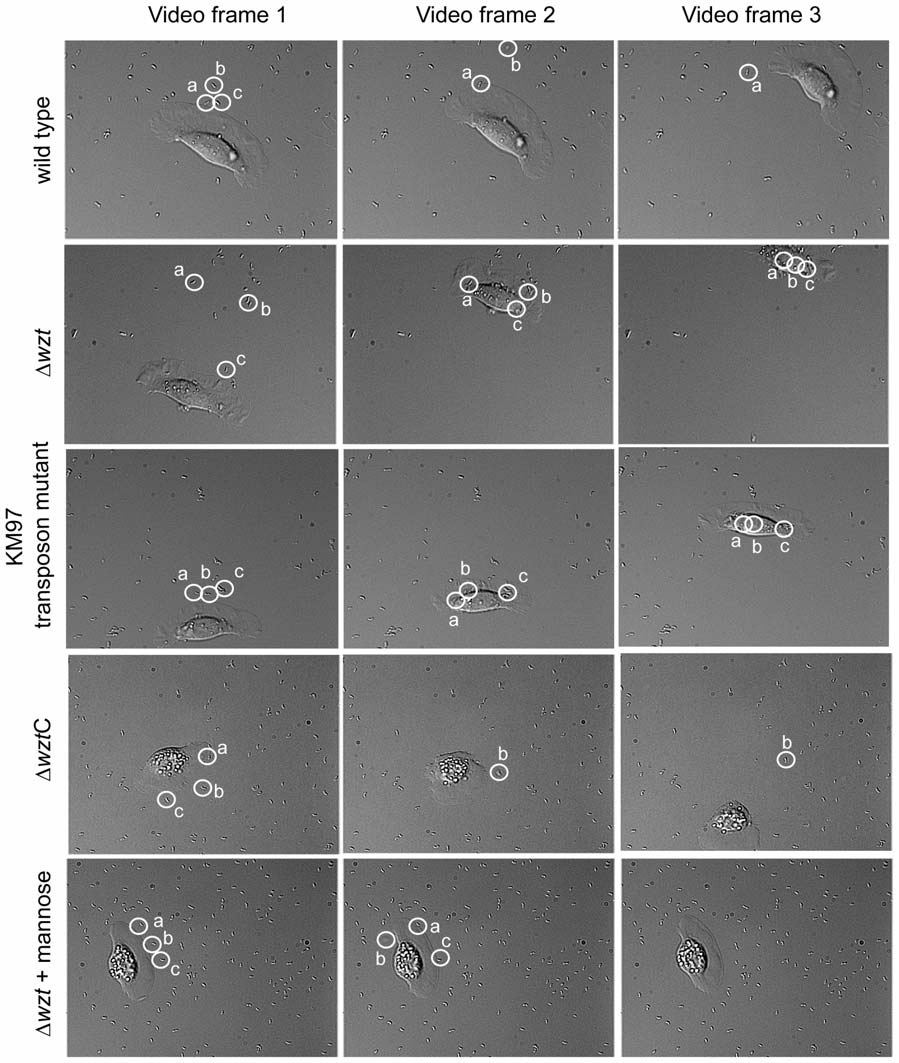
Image frames from various time points of live-cell microscopy videos of rainbow trout skin epithelial cells following infection with various *V. anguillarum* strains. Using live-cell microscopy, a video clip (Video S1, S2, S3, S6, and S7 supporting information) was recorded of the phagocytic activity of rainbow trout skin epithelial cells after infection with the wild type, the KM97 transposon mutant, the Δ*wzt* mutant, and the Δ*wzt* mutant complemented with the wild-type *wzt* gene. Three frames from each video progressing in time from left to right are shown indicating the phagocytic activity of an epithelial cell after contact with each strain. Similar videos were made for the Δ*wzm* and Δ*wbhA* mutants, and similar results to that of the Δ*wzt* mutant were seen (supporting information Videos S4 and S5). Thus, the Δ*wzt* mutant results are given as a representative for all three mutant strains. In the first frame from each video, three bacterial cells were circled and labeled. These three bacteria were followed in the two additional time frames to indicate movement of the bacteria during the movie. If the cell is no longer labeled in frames 2 and 3, then the bacterial cell was displaced from the glass surface and swam away. In the bottom set of frames, the epithelial cells were pretreated with 1 mM mannose before infection with the Δ*wzt* mutant. The three-image sets are designated by the strain mutation to the left and mutation designations followed by a C are complemented.

Epithelial cells are generally not believed to internalize microbial cells although they do have phagocytic activities to remove apoptotic bodies [19]. However, fish skin epithelial cells at the edge of a wound are proposed to phagocytize bacteria aiding wound repair by preventing infections due to opportunistic pathogens [17], [18]. *Vibrio anguillarum* proficiently colonizes the skin epithelial layer of rainbow trout and forms a biofilm-like structure [8], [9]. Colonization of the skin appears to be vital for *V. anguillarum* to cause disease. Consequently, *V. anguillarum* may encode mechanisms for evasion of phagocytosis by the fish skin epithelial cells. To test this, skin epithelial cells were isolated from rainbow trout and live-cell microscopy was used to visualize the motility of individual epithelial cells and to determine if the epithelial cells could phagocytize *V. anguillarum*. The motile epithelial cells showed the classical single, canoe-shaped lamellipodium at the leading edge that extends around most of the cell body, which is located at the back of the cell (Figure 1) [20], [21]. To test if the cells are phagocytic, latex beads (2.5 µm in diameter) were added to the epithelial cells. Live-cell microscopy showed that the epithelial cells internalized the latex beads, which were localized to the cell body resulting in cell rounding similar to what has previously been described (data not shown) [16]. To determine if *V. anguillarum* is internalized by the epithelial cells, bacteria (10^5^ cells ml^−1^) from a fresh sub-inoculation were added to the epithelial cell culture and live-cell microscopy was performed. In the resulting video (Video S1), the epithelial cells displaced the bacteria either from the surface so that they swam away or to the side so that they remained on the glass surface. The epithelial cells were also seen to migrate over a bacterial cell without disturbing it. Although a few percent of the wild-type bacteria attached to and moved along the lamellipodium to the cell body, the majority of the bacteria were not phagocytized by the epithelial cells. Figure 1 shows three frames from Video S1 at various time points showing a migrating epithelial cell as it contacts the wild-type *V. anguillarum*. Several bacteria are circled that were in contact with the lamellipodium but were not internalized as they either swam away or were pushed aside on the glass surface. These data indicate that *V. anguillarum* evades phagocytosis and likely encodes gene products utilized in an evasion mechanism.

### Identification of a DNA locus involved in the evasion of phagocytosis by rainbow trout skin epithelial cells

To identify genes involved in evading phagocytosis, a previously described mini-Tn*5phoAcm* transposon mutant library [8] was screened using live-cell microscopy for a mutant unable to evade phagocytosis by the skin epithelial cells. Each mutant was used to infect an epithelial cell culture. One mutant, KM97, was internalized by a migrating epithelial cell and the data are shown in Video S2 and Figure 1. When the migrating epithelial cell came in contact with the KM97 mutant at the leading edge of the lamellipodium, increased membrane ruffling was observed and most bacteria adhered to the epithelial cell and were transported along the lamellipodium to the cell body and internalized. This is in strong contrast to what was observed in the presence of the wild type.

**Figure 2:**
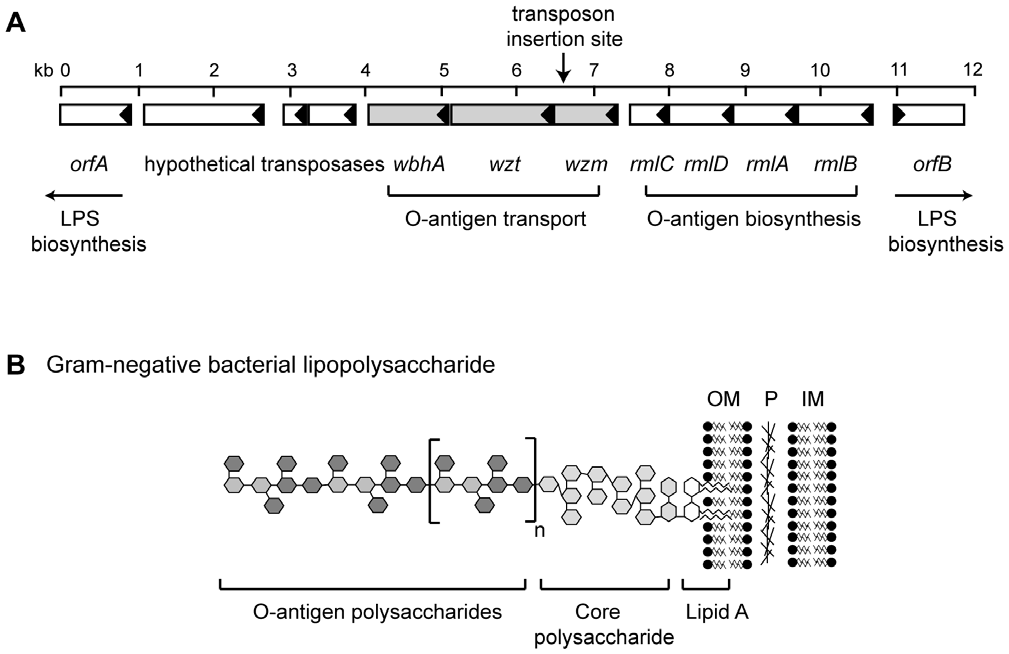
Genetic organization of the O-antigen biosynthesis gene cluster containing *wzm-wzt-wbhA*. (A) The genetic locus was identified from the draft genome sequence of the NB10 *V. anguillarum* strain (unpublished data, E. Hjerde, D.L. Milton, and N.P. Willassen). Each open reading frame (Orf) is indicated by a box and the direction of transcription is indicated by an arrowhead within each box. Grey boxes indicate the genes under investigation in this study, which are suggested to be involved in the transport of O-antigen polysaccharide. *wbhA* encodes a putative S-adenosylmethionine methyltransferase and *wzt* and *wzm* encode components of an ABC-2 subfamily of polysaccharide transporters. The four *rml* genes have homology to proteins involved in the biosynthesis of dTDP-L-rhamnose, a possible O-antigen precursor [42]. *rmlC* encodes a dTDP-6-deoxy-D-glucose-3,5-epimerase; *rmlD* encodes dTDP-6-deoxy-L-mannose dehydrogenase; *rmlA* encodes glucose-1-phosphate thymidylyltransferase; *rmlB* encodes dTDP-glucose 4,6-dehydratase. OrfA and OrfB are both part of two putative operons that encode proteins likely involved LPS biosynthesis. Orfs downstream of *wbhA* encode two putative transposases and a hypothetical protein. (B) A cartoon image of the structure of Gram-negative lipopolysaccharide. The lipopolysaccharide is a component of the outer membrane in Gram-negative bacteria and is composed of lipid A, core polysaccharide, and the O-antigen, which is a repeating sugar unit. OM, outer cell membrane; P, peptidoglycan; IM, inner membrane.

In the KM97 mutant, the transposon was localized to the *wzm* gene, which is the first gene of a putative three gene operon containing *wzm-wzt-wbhA* (Figure 2A). The putative operon lies within an O-antigen polysaccharide biosynthesis gene cluster (unpublished genome sequence of the NB10 *V. anguillarum* strain, E. Hjerde, D.L. Milton, and N.P. Willassen) [22]. Figure 2B shows a cartoon in which the O-antigen polysaccharide is shown as one of the three main components of lipopolysaccharide found on the outer surface of Gram-negative bacteria. The *wzm* and *wzt* genes are found in numerous O-polysaccharide biosynthesis gene clusters [23]. In *Escherichia coli*, Wzm and Wzt make up an ATP-binding cassette (ABC) transporter that shares similarities to the ABC-2 subfamily of ABC transporters that are conserved in many Gram-negative bacteria and that are shown so far to transport only unbranched O-polysaccharides [23], [24]. Wzm is an integral membrane protein that contains an average of six membrane-spanning domains. Wzt contains an ATP-binding motif or a Walker box and Wzt proteins associated with O-antigen biosynthesis have an extended C-terminal domain that determines substrate specificity [25]. In *V. anguillarum*, the Wzm protein is predicted to contain six transmembrane domains, which were determined by the TMHMM Server v. 2.0 at the Center for Biological Sequence Analysis at the Technical University of Denmark. Using the Blast search program at NCBI, the Wzt protein of *V. anguillarum* was shown to contain an extended C-terminus while the N-terminus contains an ATP-binding site, an ABC transporter signature motif, and a Walker box. The *wbhA* gene [26] encodes a hypothetical protein that contains an S-adenosylmethionine-dependent methyltransferase motif that was determined by a Blast search at NCBI. In *E. coli* serotype O8 and O9a, a *wbdD* gene, which encodes a methyltransferase, is found downstream of *wzm* and *wzt* homologues within a locus encoding O-antigen polysaccharide assembly systems [24]. The WbdD methyltransferase is involved in chain length determination of the lipopolysaccharide O-antigen, which is coupled to export of the O-antigen polysaccharide using the Wzm-Wzt ABC transporter dependent pathway [23], [27]. Consequently, WbhA, together with Wzm and Wzt, may be predicted to function in O-antigen polysaccharide biosynthesis.

### The Wzm-Wzt ABC transporters and the methyltransferase WbhA are essential for biosynthesis of O-antigen polysaccharide

**Figure 3:**
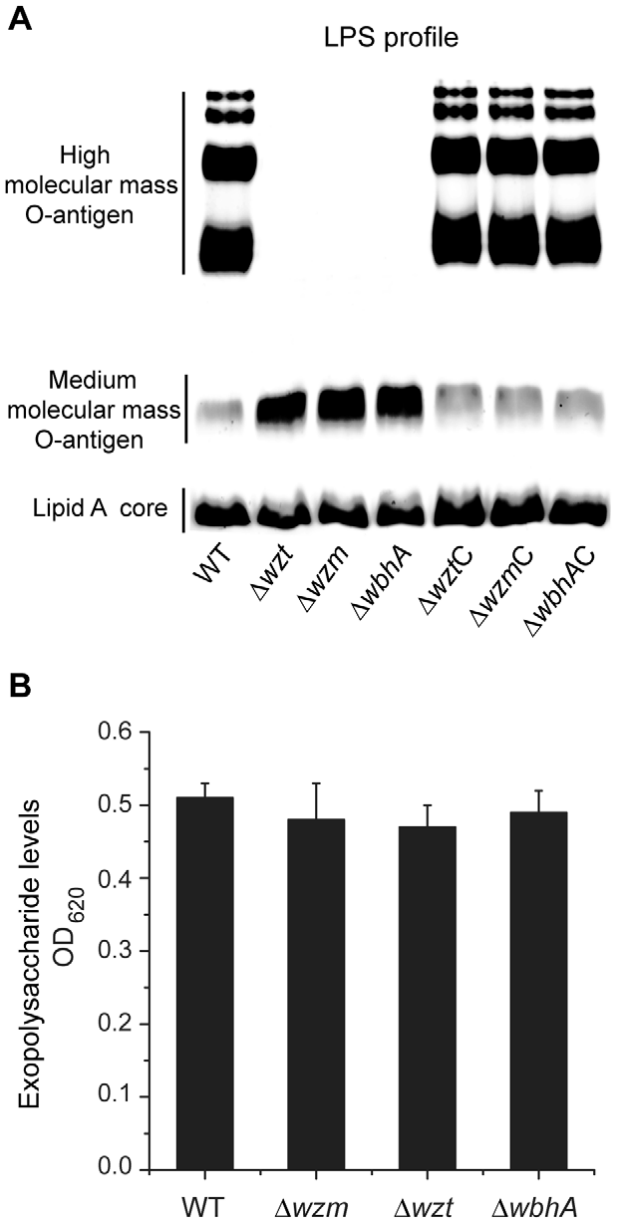
Lipopolysaccharide and exopolysaccharide analyses. (A) Lipopolysaccharides were fractionated by 15%-SDS-PAGE and detected using the Pro-Q® Emerald 300 Lipopolysaccharide Gel Stain Kit (Invitrogen). (B) Exopolysaccharides were extracted from culture supernatants and quantified using an Alcian blue technique described by Plante [56]. The amount of EPS is given as an OD_620_ reading. For both (A) and (B), strains are designated by their mutation. Mutation designations followed by a C are complemented with the respective wild-type gene.

To determine if Wzm, Wzt, and WbhA play a role in O-antigen polysaccharide biosynthesis, in-frame mutations were made that deleted the genes encoding these proteins and each mutant was tested for LPS production. All three (*Δwzm*, *Δwzt*, and *ΔwbhA*) mutants showed a loss of the O-antigen polysaccharides (Figure 3A). Complementation of each mutation with the respective wild-type gene regained the wild-type level of O-antigen polysaccharides. As group 2 capsular polysaccharides in *E. coli* are also exported by an ABC transporter similar to Wzm and Wzt [24], each mutation was also tested for the loss of exopolysaccharide. Figure 3B shows that the exopolysaccharide levels were similar to that of the wild type. Thus, Wzm, Wzt, and WbhA are essential for O-antigen polysaccharide biosynthesis but not for production of exopolysaccharides.

### O-antigen polysaccharides are required for *V. anguillarum* to evade phagocytosis by the rainbow trout skin epithelial cells

The data so far suggest that the O-antigen polysaccharides are required for *V. anguillarum* to evade phagocytosis by the migrating epithelial cells. To confirm this notion, the *Δwzm*, *Δwzt*, and *ΔwbhA* mutants were tested for their ability to evade phagocytosis by the epithelial cells. Skin epithelial cell cultures were infected with each mutant (10^5^ bacteria ml^−1^) and migrating epithelial cells were visualized by live-cell microscopy, Video S3, S4, and S5). As for the KM97 transposon mutant, all three (*Δwzm*, *Δwzt*, and *ΔwbhA*) mutants were phagocytized by the epithelial cells. When bacteria and epithelial cell came into contact at the leading edge of the lamellipodium, the bacteria attached to and migrated along the lamellipodium to the cell body where they were internalized. In addition, an increase in membrane ruffling of the lamellipodium occurred in the presence of the mutant strains as compared to contact with the wild type. Figure 1 shows frames at various time points from the video of the *Δwzt* mutant. Since all three mutants gave similar results with the live-cell microscopy, only one set of video frames is shown as a representative for all mutants. Complementation of each mutation with the respective wild-type gene re-gained the wild-type phenotype of evading phagocytosis by the epithelial cells. Figure 1 and Video S6 (supporting information) are presented as representative images for all three mutants.

**Figure 4:**
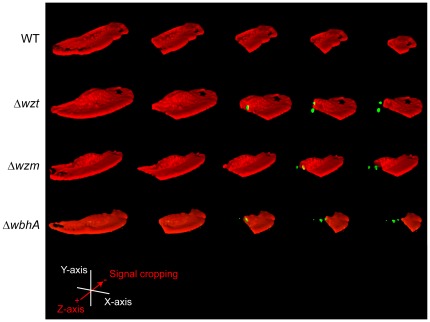
Confocal microscopy 3D-imaging of rainbow trout skin epithelial cells following infections. Skin epithelial cells were infected with the wild type and the Δ*wzt*, Δ*wzm*, and Δ*wbhA* mutants. Following fixation, the epithelial cells were visualized by labeling actin with Alexa Fluor 568 phalloidin (red). After permeabilization of the epithelial cell, bacteria were labeled using a whole-cell antiserum and a FITC-conjugated Donkey anti-rabbit IgG (green). Using the NIS-elements AR 3.2 software, a 3D rendering of a confocal microscopy z-stack covering an entire cell was done and is presented as the left most image for each strain. In each image, the phalloidin (red) signal was progressively cropped along the z-axis to reveal internal bacteria. Strains are designated by their mutations and the designations are given to the left of the image.

To confirm that the bacteria were internalized, confocal 3D-imaging microscopy was used to measure internalization and to localize the bacteria inside the cell body of the epithelial cells. Figure 4 gives a three dimensional image of a motile epithelial cell following infection by the wild type and the *Δwzm*, *Δwzt*, and *ΔwbhA* mutants. In this image, the epithelial cells are fluorescently labeled using phallodin (red), which binds to the actin filaments, and the bacteria are labeled with a FITC-labeled secondary antibody (green), which binds antibodies bound to the cell body of *V. anguillarum*. To visualize the bacteria in the cell body, a 3D rendering of a confocal microscopy z-stack covering an entire cell was done followed by progressively cropping the phalloidin signal along the Z-axis of the epithelial cell. As seen in Figure 4, the green signal or bacteria are clearly located within the cell body of the epithelial cells that were infected with the *Δwzm*, *Δwzt*, and *ΔwbhA* mutants but not in the cell infected with the wild-type strain.

**Figure 5:**
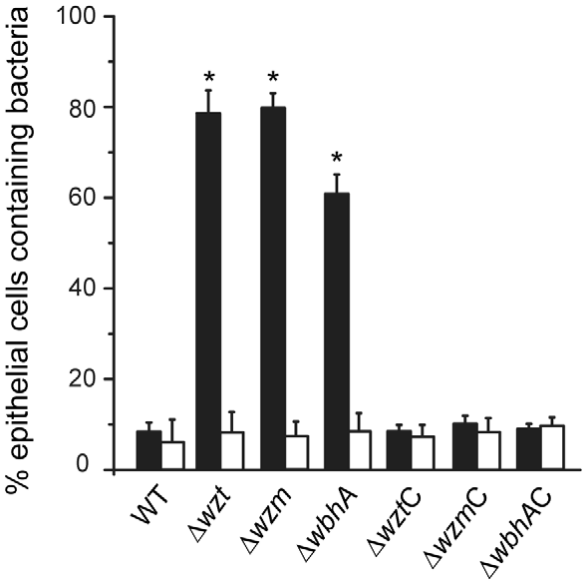
Quantification of phagocytic activity by rainbow trout skin epithelial cells following infection with various *V. anguillarum* strains. Epithelial cells were isolated from the skin of rainbow trout and either left untreated (closed bars) or pretreated with 1 mM mannose (open bars) before infection with 10^5^ bacteria ml^−1^. Following a 3 h infection, the epithelial cells were fixed and the epithelial cells and bacteria were fluorescently labeled. Confocal 3D-imaging microscopy was done on at least 100 single epithelial cells from three separate experiments to determine the number of cells that contained bacteria. These data are presented as a percent. The *V. anguillarum* strains that were tested are designated by their mutation. Mutation designations followed by a C are complemented with the respective wild-type gene. Asterisks indicate a p-value of <0.05 as determined by the Student's t-test.

To quantify the frequency of internalization of the wild type versus the three mutants, confocal 3D-imaging microscopy was used to determine the percentage of epithelial cells containing internalized bacteria (Figure 5). Following infection with the wild type, very few epithelial cells contained bacteria; whereas, the majority of epithelial cells contained bacteria when infected with any one of the mutants. Complementation of each mutation with the corresponding wild-type gene lead to a very low number of epithelial cells containing bacteria as was seen for the wild type. Taken together, these data clearly demonstrate that skin epithelial cells selectively phagocytize bacteria and that *V. anguillarum* uses its O-antigen polysaccharide to evade phagocytosis.

### Mannose abolishes phagocytosis of the O-antigen mutants by the rainbow trout skin epithelial cells

Phagocytosis usually involves pattern-recognition receptors, which mediate internalization of foreign particles by binding to molecules found on the surface of the particle [19]. To confirm further that the epithelial cells do internalize the *V. anguillarum* mutants via a receptor-mediated mechanism, we wondered if phagocytosis could be abolished by pre-treating the cells with a ligand before infection. C-type lectins are eukaryotic carbohydrate-binding proteins that mediate the recognition of bacterial pathogens [28]. Recent studies show that fish skin tissues contain mannose-binding lectins [29]–[32]. In addition, mannose is known to inhibit agglutination activity of *V. anguillarum* [33]. Thus, we wondered if skin epithelial cells utilize a mannose-binding-type lectin for internalization of the *Δwzm*, *Δwzt*, and *ΔwbhA* mutants. To reveal this, the epithelial cell culture was pretreated with 1 mM mannose before infection with the three mutant strains and the infections were followed by live-cell microscopy (Video S7). Pretreatment of the epithelial cells with mannose decreased membrane ruffling and phagocytosis of all three mutants in comparison to similar infections that used untreated epithelial cells. A video clip showing the *Δwzt* mutant as a representative for all three mutants is presented as supporting information and frames from Video S7 are presented in Figure 1. Quantification of phagocytosis after mannose treatment using confocal 3D-imaging microscopy further showed that mannose pretreatment of the epithelial cells prevented phagocytosis (Figure 5). These data suggest that a receptor-mediated mechanism is utilized by the skin epithelial cells to internalize the *V. anguillarum* mutants.

### O-antigen polysaccharides are essential for *V. anguillarum* to colonize rainbow trout skin

**Figure 6:**
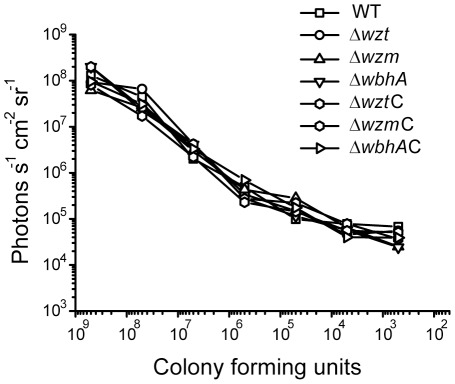
Correlation of photon emission with the number of *V. anguillarum* cells. *Vibrio anguillarum* strains carrying pNQFlaC4-lac::lux on the chromosome were grown to 5×10^8^ cells ml^−1^ and serially diluted in a black 96-well plate. Cfu counts were determined for each culture. A bioluminescent image of the 96-well plate was taken using an IVIS® SPECTRUM system (Xenogen, Caliper Life Sciences) and photon units (photons sec^−1^ cm^−2^ steradian^−1^) emitting from each well were determined. Strains are designated by their mutation and mutation designations followed by a C are complemented.

Previously, *V. anguillarum* was shown to proliferate rapidly and abundantly on rainbow trout skin forming micro-colonies that resemble biofilms [8], [9]. We wondered if the O-antigen polysaccharides play a critical role in preventing the removal of *V. anguillarum* from the fish skin by the epithelial cells. To test this, *in vivo* bioluminescent imaging was used to follow the spatial and temporal progression of a *V. anguillarum* infection in the whole rainbow trout animal. As this method detects the emission of light from host cells or tissues with a minimal background signal [34], visualization of *V. anguillarum* in the fish requires that the bacteria emit light. Thus, the wild type and the *Δwzm*, *Δwzt*, and *ΔwbhA* mutants were engineered to produce light constitutively by recombining a *lac::luxCDABE* promoter-operon fusion into an intergenic region of the chromosome as was done in a previous study [9]. Since the production of light is energetically costly for the bacterium and may affect the virulence of the bacterium [35], growth curves of the non-luminescent and luminescent strains were compared for growth differences and no significant differences were seen (data not shown). In addition, photon emission cfu^−1^ was estimated for each strain indicating an approximate equal light emission from all strains (Figure 6).

**Figure 7:**
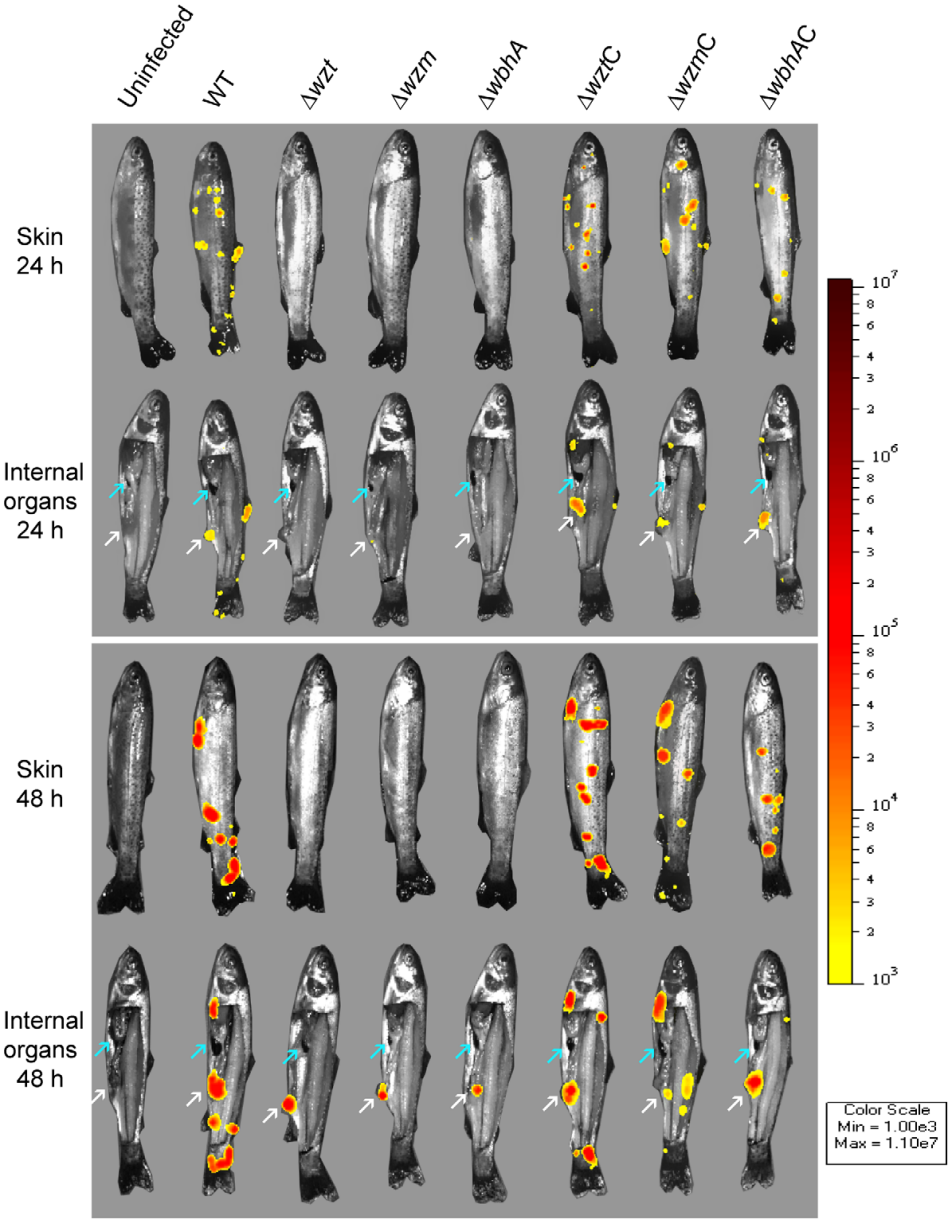
Bioluminescent imaging of *V. anguillarum* infections in rainbow trout. Rainbow trout (20 g) were infected with *V. anguillarum* strains carrying pNQFlaC4-lac::lux by bathing in infected seawater. At 24 h and 48 h, fish were sacrificed and images of the fish were obtained as described in Materials and Methods. To image internal organs, one side of the fish was removed after the skin was imaged. To measure photons from the intestines, the contents were pushed out the anus and are indicated by a white arrow. A blue arrow indicates the spleen. Strains are designated by their mutation. Mutation designations followed by a C are complemented.

The *V. anguillarum* strains carrying the *lux* operon were used to infect rainbow trout (20 g weight) by immersion into seawater containing 10^4^ bioluminescent bacteria ml^−1^. At 24 h and 48 h post infection, fish were analyzed for bioluminescent bacteria. Analyses after the 48 h time point were not done since virulent *V. anguillarum* strains cause a septicemia making it difficult to determine tissue colonization as the whole fish emits light [9]. In addition, the skin pigment of the rainbow trout blocked detection of light emission from the internal organs. Thus, one side of the fish was removed to image the internal organs after imaging both sides of the whole animal. The light that penetrated from the host tissue was captured as photons, which were used to determine a relative quantification of bacteria during the infection process. Figure 7 shows examples of the *in vivo* bioluminescent imaging of the external surfaces and the internal organs at 24 h and 48 h post infection for each strain. Table 1 summarizes the frequency of bacterial colonization of the skin, intestines, and spleen of all fish used in the infections at both 24 h and 48 h post infection. Uninfected fish that were treated similarly did not emit light. The wild type colonized the fish skin at random sites on 95% and 100% of the fish at 24 h and 48 h post infection, respectively. However, the *Δwzm*, *Δwzt*, and *ΔwbhA* mutants were never detected on the fish skin. Complementation of the mutation with the corresponding wild type gene regained the wild-type phenotype. The internal organs were also analyzed. To enhance detection of bacteria in the intestines, the faecal content was pushed out towards the anus. The wild type was detected in 85% and 95% of the intestines at 24 h and 48 h post infection, respectively. However, the mutant strains were slower than the wild type to colonize the intestines in the first 24 h (10–35%) but by 48 h, the mutant strains colonized the intestines (80–95%) as well as the wild type. The delay in colonization of the intestines by the mutant strains at 24 h could be complemented with the respective wild-type gene. Bacteria were only detected in the spleen of one fish infected with the wild type at the 48 h time point. These data indicate that the O-antigen polysaccharide is required for colonization of the fish skin and one role for the O-antigen polysaccharide may be to protect *V. anguillarum* from the phagocytic activities of the skin epithelial cells during an infection.

**Table 1:** Colonization frequency of the skin, intestines, and spleen in each infected fish group.

		Colonized fish/total fish					
		Skin		Intestines		Spleen	
Strain	Genotype^a^	24 h	48 h	24 h	48 h	24 h	48 h
Uninfected		0/20	0/20	0/20	0/20	0/20	0/20
NB10	WT	19/20	20/20	17/20	19/20	0/20	1/20
KL18	Δ*wzt*	0/20	0/20	4/20	19/20	0/20	0/20
KL19	Δ*wzm*	0/20	0/20	7/20	16/20	0/20	0/20
KL20	Δ*wbhA*	0/20	0/20	2/20	19/20	0/20	0/20
KL18c	Δ*wzt* complemented	18/20	19/20	18/20	19/20	0/20	0/20
KL19c	Δ*wzm* complemented	19/20	19/20	19/20	19/20	0/20	0/20
KL20c	Δ*wbhA* complemented	19/20	20/20	20/20	20/20	0/20	0/20

a All strains contain pNQFlaC4-lac:lux, which carries the *luxCDABE* operon and is integrated into the chromosome.

### O-antigen polysaccharides protect against antimicrobial peptide and lysozyme activities

**Figure 8:**
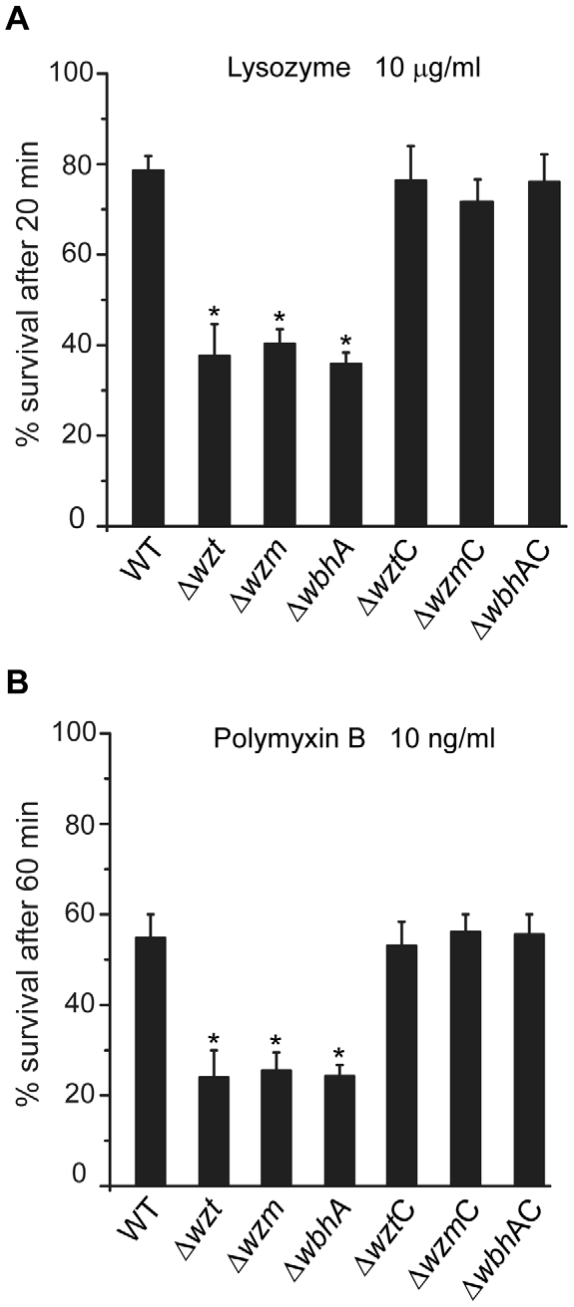
Sensitivity of *V. anguillarum* strains to antimicrobial products. Cultures of *V. anguillarum* grown to an OD_600_ of 0.2 (10^8^ cells ml^−1^) were diluted 10-fold in PBS containing either (A) 10 µg ml^−1^ of lysozyme or (B) 10 ng ml^−1^ of polymyxin B sulfate. Bacterial survival was measured at room temperature by determining the cfu in each sample at 0 min and 20 min for lysozyme and 0 min and 1 h for polymyxin B sulfate. Percent survival is the average of three experiments. Asterisks indicate a p-value of <0.05 as determined by the Student's t-test. Strains are designated by their mutation. Mutation designations followed by a C are complemented.

Studies suggest that the LPS O-antigen polysaccharides provide protection against antimicrobial factors, such as antimicrobial peptides [36]. The mucus layer covering the fish skin contains antimicrobial factors such as lysozyme and antimicrobial peptides that play a role in the innate immune defense of the skin [37]. Thus, the *Δwzm*, *Δwzt*, and *ΔwbhA* mutants were tested for a decreased resistance to lysozyme and polymyxin B compared to the wild type (Figure 8). Lysozyme hydrolyses the glycosidic bonds found in peptidoglycan; whereas, polymyxin B, an effective, commonly used antimicrobial peptide leads to membrane permeabilization. For lysozyme (10 µg ml^−1^), 78% of the wild type survived a 20 min exposure; whereas, only 40% of all three mutants survived the same treatment. For polymyxin B (10 ng ml^−1^), a 57% survival was seen for the wild type after a 1 h exposure; while, the three mutants showed only 25% survival after the same treatment. Complementation of each mutation with the corresponding wild-type gene resulted in wild-type survival rates. Hence, O-antigen polysaccharides also protect the bacterial cells from antimicrobial agents found in fish skin mucus.

## Discussion

Most reports on bacterial attachment to mucosal tissues of fish are indirect, descriptive *in vitro* studies that have been done using cell lines [3]. However, two recent *in vivo* studies give direct evidence that *V. anguillarum* forms biofilm-like microcolonies within the skin mucosal tissues of rainbow trout where it proliferates rapidly during the early stages of infection [8], [9]. During growth on the skin tissues, *V. anguillarum* is believed to produce extracellular products such as proteases, which may damage skin tissues [38], providing fast access to the blood leading to septicemia and death of the host animal. This study aimed to further our understanding of the immune defenses of the rainbow trout skin tissues and the mechanisms used by *V. anguillarum* to evade these host defenses enabling it to colonize the fish skin tissues.

Fish skin is a formidable barrier for bacterial pathogens to penetrate partly due to the numerous immune defenses associated with the skin tissues [10], [37]. The highly motile skin epithelial cells from Atlantic Salmon (*Salmo salar*) have previously been observed to internalize bacteria during wound healing and the uptake mechanism appears to be discriminatory as the epithelial cells do not phagocytize all bacteria [16]–[18]. Even though epithelial cells have the capacity for phagocytosis as they ingest apoptotic bodies during cellular turnover, they have been described as being incapable of internalizing microorganisms [19]. Using bacterial genetics together with live-cell and confocal 3D-imaging microscopy, our studies unequivocally show that single, migrating skin epithelial cells from rainbow trout (*Oncorhynchus mykiss*) efficiently phagocytize bacteria in a selective manner most likely by utilizing a phagocytic pattern-recognition receptor that binds mannose.

Phagocytosis is the cellular uptake of large particles, such as microorganisms, via a receptor-mediated process. To counteract this host defense, bacterial pathogens have evolved numerous strategies to evade phagocytosis and to resist phagocyte killing [39]. In the present study, O-antigen polysaccharides aided *V. anguillarum* in the evasion of phagocytosis by rainbow trout epithelial cells. This is a new role for LPS in the virulence of *V. anguillarum.* The O-antigen polysaccharide of *V. anguillarum* was previously shown to be essential for virulence in fish animal models, for resistance to complement-mediated killing found in fish blood serum, and for anguibactin-mediated iron transport [40]–[43]. Our study suggests that the O-antigen masks putative mannose structures on the surface of the bacteria that are recognized by receptors on the surface of the skin epithelial cells. One possible receptor type is the C-type mannose-specific lectin [28]. Mannose-binding lectins may also bind glucose, L-fucose, N-acetyl-mannosamine and N-acetyl-glucosamine. All of which are sugar residues found on bacterial cell surfaces. Fish skin mucus from various species of fish is described to contain a diverse array of lectins, including C-type lectins [44]. Recent studies show that fish skin tissues, including that of rainbow trout (*Oncorhynchus mykiss*), express mannose-binding lectins [29]–[31]. In one study, a mannose-specific lectin is shown to be associated with fish skin epithelial cells [32].

An antiphagocytic role has been described for the O-antigen associated with other pathogens, *E. coli, Salmonella enterica* serovar Typhimurium, *Burkholderia cenocepacia*, *Neisseria gonorrhoeae*, and *Haemophilus ducreyi* [45]–[49]. In *E. coli*, the O-antigen is suggested to mask the core region of the LPS, which binds the unique C-type lectin DC-SIGN (dendritic cell specific intercellular adhesion molecule nonintegrin) associated with phagocytic dendritic cells [28], [45]. A genome analysis of C-type lectin domains in the puffer fish *Fugu rubripes* identified all but two groups of C-type lectin domains identified in mammals and the DC-SIGN subgroup is overrepresented [50]. Although an analysis for DC-SIGN receptors has not been done using rainbow trout, the puffer fish studies may suggest that the rainbow trout skin epithelial cells express a DC-SIGN-like receptor that is involved in the phagocytosis of the *V. anguillarum* O-antigen transport mutants.

*Vibrio anguillarum* colonizes quickly and efficiently the skin tissues of rainbow trout during the early stages of infection [8], [9]. The fish skin mucosal layer is an important source of nutrients enabling the bacterium to survive in an otherwise nutrient poor environment. Colonization of the skin tissues greatly aids disease development to a late stage septicemia. Our study is one of the first to provide direct insight into a mechanism that many bacteria may use to colonize the fish skin epidermal mucosa. The highly motile rainbow trout skin epithelial cells play a vital role in the immune defense associated with the skin tissues by phagocytizing bacteria and keeping the skin clear of potential pathogens. To colonize fish skin tissues, other bacteria, like *V. anguillarum*, may utilize LPS O-antigen to mask surface-located molecular patterns that are recognized by receptors on the phagocytic skin cells. Understanding bacterial mechanisms required for colonization of fish skin tissues is vital to provide better insight into how bacteria are transmitted from fish to fish or from fish to human within aquaculture or in the wild and to develop improved therapeutics for the prevention of fish and/or human disease.

## Materials and Methods

### Ethics statement

The infection studies were performed in strict accordance with the Swedish Bioethical Guidelines for care and use of laboratory animals. The protocol was approved by the Umeå Committee on the Ethics of Animal Experiments (Permit Number: A-103-11).

### Bacterial strains, plasmids and culture conditions

Bacterial strains and plasmids used in this study are given as supplementary information in Table S1. *V. anguillarum* strains were routinely grown in Trypticase soy broth containing 1% sodium chloride (TSB-1%) at 24°C with aeration, or on Trypticase soy agar (TSA) grown at room temperature. *Escherichia coli* was routinely grown at 37°C with aeration in Luria broth (per liter: Bacto Tryptone, 10 g; Bacto yeast extract, 5 g; sodium chloride, 5 g). Plasmid transfers from *E. coli* to *V. anguillarum* were performed as described previously [51]. The vibrio selective medium TCBS agar (Difco) containing 10 µg ml^−1^ chloramphenicol was used after conjugation to select against *E. coli*.

### PCR conditions, sequencing, and DNA techniques

PCR was performed as previously described [52]. When a PCR fragment required minimal errors, the high-fidelity KOD polymerase (Novagen) was used. Unless otherwise stated, conditions for various DNA techniques are according to Sambrook *et al*. [53]. Reaction conditions for DNA-modifying enzymes and DNA restriction enzymes were performed according to the manufacturer's instructions. Genomic DNA sequencing was done by Eurofins MWG GmbH as part of a genome sequencing project (unpublished data; E. Hjerde, D.L. Milton, and N.P. Willassen).

### Accession number

The DNA sequence data for the *wzm-wzt-wbhA* operon have been submitted to the DDB/EMBL/GeneBank databases under accession number JQ920376.

### In-frame deletion mutagenesis

In-frame, single gene deletions of the *wzm*, *wzt*, and *wbhA* genes were made by allelic exchange using the R6K origin-based suicide vector pDM4 as described previously [51]. A null mutant allele of each gene was created by overlap PCR fusing several codons from both the 5′ and 3′ ends of each gene (Table S1). Each allele was cloned into pDM4 creating plasmids pDM4-wzt-AD, pDM4-wzm-AD, and pDM4-wbhA-AD. The plasmids were mobilized into the wild type and after allelic exchange, the mutants KL18 (Δ*wzt*), KL19 (Δ*wzm*), and KL20 (Δ*wbhA*) were created. All mutations were confirmed by sequencing a DNA fragment that was amplified by PCR from the mutated chromosomal locus in each mutant strain. Primers used in the overlap PCR to make each allele are included as supplementary information in Table S2. For complementation of each mutation, reverse allelic exchange was done. The wild-type genes were amplified using the primer A and primer D that was used to make the mutant allele and cloned into pDM4 creating pDM4-wzm-wt, pDM4-wzt-wt, and pDM4-wbhA-wt. Each plasmid was mobilized into the respective mutant strain and reverse allelic exchange was performed as described for the allelic exchange.

### LPS and EPS analyses

Lipopolysaccharides were isolated from each bacterial strain by a Proteinase K digestion of whole-cell lysates as described by Preston and Penner [54]. The LPS were fractionated by 15%-SDS-PAGE [55] and visualized using Pro-Q® Emerald 300 Lipopolysaccharide Gel Stain Kit (Invitrogen) according to the manufacturer's instructions and an ImageQuant LAS4000 biomolecular imager.

Exopolysaccharides were quantified from spent culture supernatants of each strain using an Alcian blue technique described by Plante [56]. Bacterial cultures (10 ml) were grown for three days in TSB-1% at 24°C with aeration. After bringing the bacterial cultures to equal cell densities, cells from 1 ml of culture were pelleted using low speed centrifugation (12,000 g, 15 min, 4°C) and the supernatants were collected and used to quantify the EPS.

### Isolation of rainbow trout skin epithelial cells

Using a fine tip forceps, single scales were picked from the skin of a 20 g rainbow trout (*Oncorhynchus mykiss*) and placed in a small petri dish containing Hank's balanced salt solution (HBSS, Invitrogen) with PEST (Invitrogen) at a final concentration of 100 U of penicillin and 100 µg of streptomycin. To remove skin mucus, the scales were washed 5 times in HBSS containing PEST. For confocal 3D-imaging microscopy, a drop of HBSS containing PEST was spotted onto a collagen I coated coverslip (BD Biosciences) that was placed in a small petri dish and 5 fish scales were equally distributed within the buffer solution on the coverslip. A second coverslip of larger size was placed on top of the fish scales sandwiching them between the two coverslips. Additional drops of HBSS with PEST were added to the edges of the coverslips to prevent drying out. The fish scales were then incubated overnight at 12°C to allow the epithelial cells to migrate from the fish scale surface. For live-cell microscopy, glass bottom microwell dishes (MatTek) were pretreated with poly-L-lysine, which provided a sticky surface for highly motile *V. anguillarum* and enhanced visualization of bacterial-host interactions using the live-cell microscope. For poly-L-lysine pretreatment, 300 µl of a 0.01% poly-L-lysine solution (70,000–150,000 mol. wt.) was placed on the glass bottom of a microwell dish. The microwell dish was incubated overnight at room temperature followed by 3 washings with phosphate buffered saline (PBS). Following the PBS washings, 100 µl HBSS with PEST were spotted into the poly-L-lysine treated glass bottom and 5 scales were equally distributed on the glass surface. A coverslip larger than the microwell was placed on top of the scales to apply pressure that forces the scales towards the glass bottom. An additional 1.4 ml of HBSS and PEST was added to the microwell dish, which was then incubated overnight at 12°C to allow the epithelial cells to migrate from the fish scale surface. These epithelial cell preparations were then used in live-cell microscopy or confocal 3D-imaging microscopy.

### Live-cell microscopy

Live cell microscopy was used to visualize motility and phagocytic activity of the rainbow trout skin epithelial cells. Cells isolated in the glass-bottom microwell dishes were used. The top coverslip was removed and the epithelial cells were gently washed 3 times in HBSS to remove PEST prior to infection with bacteria. After washing, the epithelial cells were overlaid with 1 ml of HBSS and infected with bacteria. Prior to infection, bacterial strains were grown overnight at 24°C in TSB-1% with aeration, sub-inoculated as a 1/10 dilution in TSB-1%, and incubated for an additional 3 hours in TSB-1%. From each bacterial culture, an equivalent of 10^8^ cells were removed (OD_600_ of 0.2 equals 1×10^8^ cells ml^−1^), pelleted in a microfuge, washed, and resuspended in 1 ml of HBSS. To infect the epithelial cell culture, 1 µl of each bacterial sample in HSBB was added to a microwell containing epithelial cells giving a final concentration of 1×10^5^ bacteria ml^−1^. Following the addition of bacteria, single, motile epithelial cells were visualized using an inverted Nikon Eclipse Ti live-cell microscope equipped with a Plan Apo VC 60×/1.40 oil objective. Motility and phagocytic activity of the epithelial cells were recorded for 20 min taking 30 frames per second. The bacteria-host cell association was then analyzed frame by frame of a recorded movie. All images were processed with real-time deconvolution using the NIS-Elements AR 3.2 software. For the initial experiment to demonstrate phagocytic activity of the rainbow trout epithelial cells, 2 µm-latex beads (∼10^4^ beads ml^−1^, Chemika) were used in the place of bacteria. For mannose inhibition of phagocytosis, the epithelial cells were pretreated before bacterial addition with HBSS containing 1 mM mannose for 20 min at room temperature followed by 3 washings with HBSS without mannose. All live-cell microscopy analyses were repeated in at least three-independent experiments and videos and images presented are representative of these experiments.

### Confocal microscopy

For 3D-imaging of bacteria within the epithelial cells, the epithelial cells isolated on collagen I coated coverslips were used in confocal 3D-imaging microscopy. The top coverslip was removed and the bottom coverslip with attached epithelial cells was washed 3 times in HBSS to remove PEST before infecting with bacteria. The coverslip was placed in a small petri dish containing 1 ml of HBSS. To infect the epithelial cells, bacterial cells were prepared as described above for live-cell microscopy and were added to the epithelial cells to a final concentration of 1×10^5^ bacteria ml^−1^. After 3 hours infection at room temperature, the coverslip was washed 3 times in PBS to remove unattached epithelial cells and bacteria. The cells were then fixed with 4% paraformaldehyde (pH 7.4) in PBS for 10 minutes at room temperature. After fixation, the epithelial cells were washed 3 times in PBS, permeabilized with 1 ml of PBS containing 0.5% Triton X-100 (Sigma) for 3 minutes, and washed twice in PBS. The cells were then treated with PBS containing 1% bovine serum albumin to block non-specific binding sites prior to staining the cells. To visualize internalized bacteria, a *V. anguillarum* whole-cell antiserum was added to the permeabilized epithelial cells and incubated for 30 min at room temperature. After washing twice in PBS, the cells were incubated with a FITC-conjugated Donkey Anti-Rabbit IgG antiserum (Jackson ImmunoResearch Laboratories) for 30 minutes and washed twice in PBS. To visualize the epithelial cells, actin was stained using Alexa Fluor 568 phalloidin (Invitrogen) according to the manufacturer's instructions. The coverslip with the attached epithelial cells was mounted onto a glass slide using ProLong® Gold antifade mounting reagent (Invitrogen). Epithelial cells were imaged using a Nikon D-Eclipse C1 fluorescence confocal microscope equipped with a Plan Apo VC 60×/1.40 oil objective. Epithelial cells were detected using lasers with an excitation/emission of 578/600 nm and bacteria were detected using lasers with an excitation/emission of 515/530 nm. A 3D rendering of a confocal z-stack covering the entire cell thickness using two colors and image cropping was done with the imaging software NIS-Elements EZ-C1 Ver. 3.90. All images were processed identically in Adobe Photoshop CS2. All confocal imaging analyses were repeated in at least three-independent experiments and the images presented are representative of these experiments. To quantify the number of epithelial cells with internalized bacteria, a minimum of 100 single epithelial cells from three separate experiments were analyzed by doing a 3D image stack of each epithelial cell and cropping this image as described above.

### Rainbow trout infections

Rainbow trout (*Oncorhynchus mykiss*) with an approximate weight of 20 g were infected with *V. anguillarum* strains carrying pNQFlaC4-lac::lux at 18°C by bathing in seawater containing 10^4^ bacteria ml^−1^ as described previously [51]. After 30 min, the infected seawater was removed and replaced with fresh seawater and the fish were held at 18°C. At 24 h and 48 h, the fish were euthanized by an overdose of anesthetic before visualization in the *in vivo* imaging system. Two independent infections were done using 10 fish for each infection.

### In-vivo bioluminescent imaging of *V. anguillarum* during an infection of rainbow trout

To visualize *V. anguillarum* during an infection of rainbow trout using *in vivo* bioluminescent imaging, the bacterial strains used were engineered to produce light as describe previously [9]. The R6K-origin-based suicide plasmid pNQFlaC4-lac::lux that carries the *Photorhabdus luminescens luxCDABE* operon, which is constitutively expressed in *V. anguillarum*, was recombined into an intergenic region of the chromosome of each strain. Photon emission was correlated with bacterial cell numbers by growing each strain containing pNQFlaC4-lac::lux to an OD_600_ of 0.7 (5×10^8^ cells ml^−1^) in TSB-1%, serially diluting the cells in 100 µl of the same medium in a black 96-well plate, and taking an image of the 96-well plate using an IVIS® SPECTRUM system (Xenogen, Caliper Life Sciences). Luminescent signals were collected for 10 sec, images were processed with a binning of 4, and other photographic parameters were identical for each dilution series. Photon units (photons sec^−1^ cm^−2^ steradian^−1^) emitting from each well was determined using the Living Image® 3.0 software from Xenogen. Cfu counts were determined for each culture.

Rainbow trout were infected with *V. anguillarum* strains carrying pNQFlaC4-lac::lux as described above. At 24 h and 48 h, images of the fish were obtained as described above except that luminescent signals were collected for 5 min. The skin was imaged and then one side of the fish was removed to image the internal organs as the skin pigment blocked the detection of photon emission from internal organs. To enhance detection of bacteria in the intestines, the faecal contents were pushed out the anus.

### Sensitivity to lysozyme and polymyxin B

Overnight cultures of *V. anguillarum* grown in TSB-1% at 24°C with aeration were diluted in the same medium and incubated to an OD_600_ of 0.2 (10^8^ cells ml^−1^). The bacterial cells were diluted 10-fold in PBS containing either 10 µg ml^−1^ of hen egg white lysozyme (Fluka) or 10 ng ml^−1^ of freshly prepared polymyxin B sulfate (Sigma) and incubated at room temperature. Bacterial survival was measured by determining the cfu in each sample at 0 min and 20 min for lysozyme and at 0 min and 60 min for polymyxin B. The percent survival given is the average of three experiments.

## Supporting Information

Video S1 **Live-cell microscopy video demonstrating that the wild-type** ***V. anguillarum*** **evades phagocytosis by rainbow trout skin epithelial cells.** Skin epithelial cells were isolated from rainbow trout and infected with 10^5^ bacteria ml^−1^. Following infection, phagocytic activities of motile epithelial cells were tracked using live-cell microscopy.
(MP4)

Video S2 **Live-cell microscopy video demonstrating that the** ***V. anguillarum*** **transposon mutant KM97 is phagocytized by rainbow trout skin epithelial cells.** Skin epithelial cells were isolated from rainbow trout and infected with 10^5^ bacteria ml^−1^. Following infection, phagocytic activities of motile epithelial cells were tracked using live-cell microscopy. The transposon was localized to the *wzm* gene, which is the first gene of a three-gene operon involved in LPS O-antigen transport.
(MP4)

Video S3 **Live-cell microscopy video demonstrating that the** ***V. anguillarum Δwzt*** **mutant, which is defective for O-antigen transport, is phagocytized by rainbow trout skin epithelial cells.** Skin epithelial cells were isolated from rainbow trout and infected with 10^5^ bacteria ml^−1^. Following infection, phagocytic activities of motile epithelial cells were tracked using live-cell microscopy.
(MP4)

Video S4 **Live-cell microscopy video demonstrating that the** ***V. anguillarum Δwzm*** **mutant, which is defective for O-antigen transport, is phagocytized by rainbow trout skin epithelial cells.** Skin epithelial cells were isolated from rainbow trout and infected with 10^5^ bacteria ml^−1^. Following infection, phagocytic activities of motile epithelial cells were tracked using live-cell microscopy.
(MP4)

Video S5 **Live-cell microscopy video demonstrating that the** ***V. anguillarum ΔwbhA*** **mutant, which is defective for O-antigen transport, is phagocytized by rainbow trout skin epithelial cells.** Skin epithelial cells were isolated from rainbow trout and infected with 10^5^ bacteria ml^−1^. Following infection, phagocytic activities of motile epithelial cells were tracked using live-cell microscopy.
(MP4)

Video S6 **Live-cell microscopy video demonstrating that the** ***V. anguillarum Δwzt*** **mutant carrying the wild-type** ***wzt*** **gene regains the ability to evade phagocytosis by the rainbow trout skin epithelial cells.** Skin epithelial cells were isolated from rainbow trout and infected with 10^5^ bacteria ml^−1^. Following infection, phagocytic activities of motile epithelial cells were tracked using live-cell microscopy. This video is representative of epithelial cell interactions also associated with the *Δwzm* and *ΔwbhA* mutants carrying the respective complementing wild-type gene. All three complemented mutant strains regained the ability to evade phagocytosis.
(MP4)

Video S7 **Live-cell microscopy video demonstrating that mannose blocks phagocytosis of the** ***V. anguillarum Δwzt*** **mutant by rainbow trout skin epithelial cells.** Rainbow trout skin epithelial cells were isolated and incubated with 1 mM mannose prior to infection with 10^5^ bacteria ml^−1^. Following infection, phagocytic activities of motile epithelial cells were tracked using live-cell microscopy. This video suggests that the epithelial cells utilize a mannose-binding receptor to bind sugar residues on the surface of *V. anguillarum* strains lacking the O-antigen. The O-antigen may be predicted to mask these sugar residues on the bacterial surface blocking phagocytosis.
(MP4)

Table S1 **Bacterial strains and plasmids used in this study.**
(DOCX)

Table S2 **Primer sequences.** Primers used in this study to create deletion alleles of each reference gene are listed here.
(DOCX)
