# Supplementary material for: Lipopolysaccharide O-Antigen Prevents Phagocytosis of Vibrio anguillarum by Rainbow Trout (Oncorhynchus mykiss) Skin Epithelial Cells
Source: PLoS One. 2012 May 25;7(5):e37678. doi: 10.1371/journal.pone.0037678 (PMC3360773; doi:10.1371/journal.pone.0037678)
Supplement: Table S1 — Bacterial strains and plasmids used in this study. (DOCX) [file pone.0037678.s008.docx]

**Table S1. Bacterial strains and plasmids used in this study**

| **Strain or plasmid** | **Genotype or relevant markers^a^** | **Reference/source** |
| --- | --- | --- |
| **Strains** |  |  |
| NB10 | Wild type *V. anguillarum*, serotype O1, clinical isolate from the Gulf of Bothnia | [1] |
| KM97 | Cm^r^; mini-Tn*5phoAcm*::*wzm* LPS mutant; NB10 derivative | [2] |
| KL18 | *wzt* in-frame deletion; codons 1-13 fused to codons 441-446; NB10 derivative | This study |
| KL19 | *wzm* in-frame deletion; codons 1-10 fused to codons 263-268; NB10 derivative | This study |
| KL20 | *wbhA* in-frame deletion; codons 1-11 fused to codons 360-371; NB10 derivative | This study |
| KL18c | *wzt* mutant complemented with the wild type gene via reverse allelic exchange | This study |
| KL19c | *wzm* mutant complemented with the wild type gene via reverse allelic exchange | This study |
| KL20c | *wbhA* mutant complemented with the wild type gene via reverse allelic exchange | This study |
| **Plasmids** |  |  |
| pDM4 | Cm^r^, suicide vector with an R6K origin (requires *pir*) and *sacBR* of *Bacillus subtilis* | [3] |
| pDM4-wzm-AD | Cm^r^, pDM4 carrying a *wzm* mutant allele that deletes *wzm* codons 11-262 (756 bp) | This study |
| pDM4-wzt-AD | Cm^r^, pDM4 carrying a *wzt* mutant allele that deletes *wzt* codons 14-440 (1281 bp) | This study |
| pDM4-wbhA-AD | Cm^r^, pDM4 carrying a *wbhA* mutant allele that deletes *wbhA* codons 12-359 (1044 bp) | This study |
| pDM4-wzm-wt | Cm^r^, pDM4 carrying the *wzm* wild-type gene | This study |
| pDM4-wzt-wt | Cm^r^, pDM4 carrying the *wzt* wild-type gene | This study |
| pDM4-wbhA-wt | Cm^r^, pDM4 carrying the *wbhA* wild-type gene | This study |
| pNQFlaC4-lac::lux | Cm^r^, R6K origin; a 250-bp intergenic region downstream of *flaC* and P_A1/04/03_::*luxCDABE* | [4] |

^a^ Cm^r^, chloramphenicol resistance.

**References**

1. Norqvist A, Hagström Å, Wolf-Watz H (1989) Protection of rainbow trout against vibriosis and furunculosis by the use of attenuated strains of *Vibrio anguillarum*. Appl Environ Microbiol 55: 1400-1405.
2. Croxatto A, Lauritz J, Chen C, Milton DL (2007) *Vibrio anguillarum* colonization of rainbow trout integument requires a DNA locus involved in exopolysaccharide transport and biosynthesis. Environ Microbiol 9: 370-382.
3. Milton DL, O’Toole R, Hörstedt P, Wolf-Watz H (1996) Flagellin A is essential for the virulence of *Vibrio anguillarum*. J Bacteriol 178: 1310-1319.
4. Weber B, Chen C, Milton DL (2010) Colonization of fish skin is vital for *Vibrio anguillarum* to cause disease. Environ Microbiol Rep 2: 133-139.
