# Supplementary material for: Lipopolysaccharide O-Antigen Prevents Phagocytosis of Vibrio anguillarum by Rainbow Trout (Oncorhynchus mykiss) Skin Epithelial Cells
Source: PLoS One. 2012 May 25;7(5):e37678. doi: 10.1371/journal.pone.0037678 (PMC3360773; doi:10.1371/journal.pone.0037678)
Supplement: Table S2 — Primer sequences. Primers used in this study to create deletion alleles of each reference gene are listed here. (DOCX) [file pone.0037678.s009.docx]

**Table S2. Primer sequences**

| **Primer name** | **5´-3´sequence*** | **Reference gene** |
| --- | --- | --- |
| wzm-A | GG**ACTAGT**TGCCCATTACTTTCC | *wzm* |
| wzm-B | TACATCAGCAAAATGATGGACAAACTTGAACGG | *wzm* |
| wzm-C | CATTTTGCTGATGTACTATAGGGA | *wzm* |
| wzm-D | GGT**GAGCTC**TTCCGACAGTCTCAC | *wzm* |
| wzt-A | GG**ACTAGT**AGGCTATAGAAATAC | *wzt* |
| wzt-B | GATATAATTCATTGATTTAGTTATATTCTCACA | *wzt* |
| wzt-C | TCAATGAATTATATCGACTAAATA | *wzt* |
| wzt-D | GGT**GAGCTC**TCAGAACCAATAAAA | *wzt* |
| wbh-A | GC**ACTAGT**CTATCAAGTCTGGTA | *wbhA* |
| wbh-B | TTTGTATAGCCTTGATTTAATCGTTTGGAATAT | *wbhA* |
| wbhA-C | TCAAGGCTATACAAATTAAGGTCT | *wbhA* |
| wbhA-D | GGT**GAGCTC**ATCTACAAGCAGATC | *wbhA* |

* Bold lettering in the primer sequences indicates restriction endonuclease sites.
